# Phase 1 randomized study on the safety, tolerability, and pharmacodynamic cognitive and electrophysiological effects of a dopamine D_1_ receptor positive allosteric modulator in patients with schizophrenia

**DOI:** 10.1038/s41386-020-00908-0

**Published:** 2020-11-17

**Authors:** Amit Desai, Lauren Benner, Ruishan Wu, Lev Gertsik, Paul Maruff, Gregory A. Light, Tolga Uz, Gerard J. Marek, Tong Zhu

**Affiliations:** 1 Astellas Pharma Global Development, Inc., Northbrook, IL USA; 2 California Clinical Trials Medical Group, Inc., Glendale, CA USA; 3 Cogstate, Inc., Melbourne, VIC Australia; 4 University of California, San Diego, San Diego, CA USA

**Keywords:** Drug development, Outcomes research

## Abstract

ASP4345, a novel dopamine D_1_ receptor positive allosteric modulator, is being evaluated for the treatment of cognitive impairment associated with schizophrenia (CIAS). This phase 1 multiple ascending-dose study (NCT02720263) assessed the safety, tolerability, and pharmacodynamics of ASP4345 in patients with schizophrenia/schizoaffective disorder. Pharmacodynamic assessments were Cogstate cognitive tests and electrophysiological biomarkers, including gamma-band power and phase synchronization in response to 40-Hz auditory steady-state stimulation, as well as mismatch negativity (MMN) and P3a event-related potentials. The sample size determination was based on standard practice in assessing safety and tolerability of a new chemical entity. Data were summarized by conversion of this data into effect sizes using descriptive and inferential statistics. A total of 36 randomized patients received ASP4345 (3, 15, 50, and 150 mg; *n* = 9 each dose) and 12 patients received placebo. Patients in the ASP4345 group experienced 73 treatment-emergent adverse events (TEAEs) and 34 TEAEs were reported for the placebo group. The most common TEAEs were headache and somnolence and nearly all TEAEs were mild in severity. No changes in mood or self-reports of suicidal ideation/behavior were observed. Improvements in performance on cognitive tests were noted, which suggests a potential improvement in psychomotor function and visual attention. Furthermore, positive changes in neurophysiological biomarkers (auditory steady-state response [ASSR] and MMN) suggest improvement in information processing. The findings need to be confirmed in studies with a larger patient population. Nonetheless, the trends in safety and pharmacodynamic data support further clinical development of ASP4345 for the treatment of CIAS.

## Introduction

The central role of dopamine in working memory within the nonhuman primate prefrontal cortex was discovered by demonstrating that simply depleting dopamine reprised the working memory deficits produced by complete ablation of the prefrontal cortex [1]. The cognitive role of dopamine appears to arise from the midbrain dopamine neurons that transmit multiple uniform signals to their target areas in the prefrontal cortex [2, 3]. Subsequent research findings have confirmed that dopamine and D_1_ receptors have a strong regulatory role in dorsolateral prefrontal cognitive function (i.e., cognition, emotion, and motor function) [4–6]. Disruption to dopaminergic neurotransmission is a central characteristic of schizophrenia, and while overactivity of distributed dopamine networks with influences of the dopamine D_2_-like receptor family (e.g., D_2_, D_3_, and D_4_ receptors) is putatively involved in the primary psychotic manifestations of the disease, activation of dopamine D_1_ receptors has been recommended as a target for the investigation of treatment of cognitive impairment associated with schizophrenia (CIAS) [7]. More recent work identifying increases in prefrontal cortical dopamine D_1_ receptors in antipsychotic-naive patients with schizophrenia has added further interest to physiologically relevant modification of presumed decreased dopamine in the dorsolateral prefrontal cortex of patients with schizophrenia [8]. Dopamine D_1_ agonists were previously investigated in CIAS; however, their use is limited by off-target safety concerns, particularly seizures, and orthostatic hypotension [5].

**Fig. 1:**
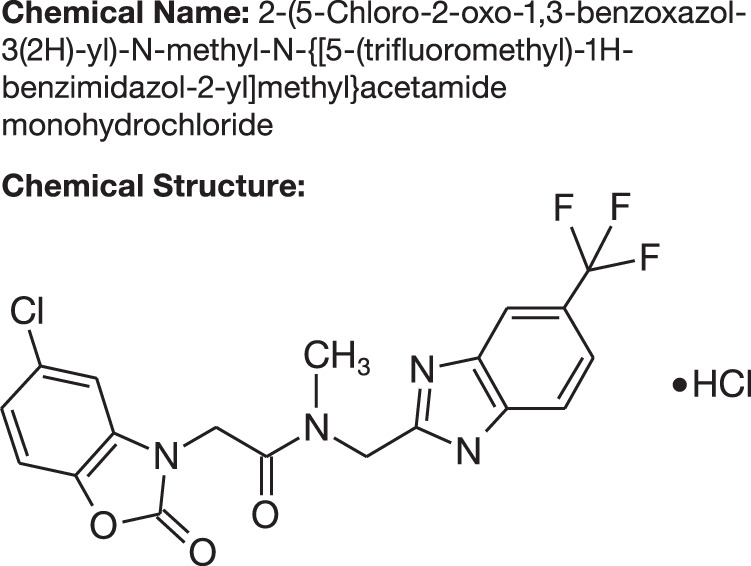
Chemical name and structure of ASP4345.

ASP4345 is a novel compound that selectively binds to, and enhances the activity of, dopamine D_1_ receptor orthosteric agonists (Fig. 1). At present, the binding site for ASP4345 is unknown. The dopamine D_1_ receptor PAM activity of ASP4345 has been shown in vitro by a twofold shift in the dopamine concentration-response curve for cyclic adenosine monophosphate accumulation [9]. In vivo data consistent with dopamine D_1_ receptor PAM activity has also been suggested from experiments with a mouse Y-maze behavioral assay [9] and using other rodent models of cognitive deficits (data on file).

As a PAM of the dopamine D_1_ receptor, ASP4345 may have fewer off-target effects because of its selectivity toward dopamine D_1_ receptors [9, 10]. Additionally, ASP4345 has the potential to effectively ameliorate CIAS without the development of commonly observed side effects associated with dopamine D_1_ agonists, such as hypotension and seizures, because it enhances ambient dopaminergic tone rather than activating dopamine D_1_ receptors independent of dopamine cell firing and dopamine release from terminals [10–12]. ASP4345 was well tolerated in animal studies and healthy volunteers, which prompted further clinical development of this compound. Moreover, two phase 1 studies showed that ASP4345 is rapidly absorbed, has a long half-life, reaches the brain (confirmed in healthy human subjects based on the ratio of CSF to total plasma for AUC_24_ and *C*_max_, as well as the ratio of CSF to unbound plasma for *C*_max_), and has a pharmacokinetic profile that is not affected by food [13]. Presented here are the results from a phase 1 multiple ascending-dose study (NCT02720263) conducted to assess the safety, tolerability, and pharmacodynamics of ASP4345 in patients with clinically stable schizophrenia or schizoaffective disorder. The pharmacokinetic data from this study are published elsewhere [13].

## Patients and methods

### Study design

This multiple ascending-dose study was a double-blind, placebo-controlled trial wherein patients were randomized 3:1 to receive ASP4345 or placebo administered as a single daily oral dose for 14 days. The ASP4345 cohorts received an initial dose of 3 mg, followed by 15, 50, or 150 mg in successive independent cohorts. The ensuing doses in each cohort were not predefined, but were decided by the sponsor and investigator/delegate based on overall exposure and differences between subsequent cohorts (i.e., predicted exposure could not exceed the mean exposure limit and dose increments could not exceed fivefold between successive cohorts). Doses were administered under fasted conditions on days 1, 7, and 14, and under fed conditions on days 2–6 and 8–13.

The study was conducted at one site in the United States, in accordance with the ethical principles based on the Declaration of Helsinki, Good Clinical Practice, International Council for Harmonization guidelines, and applicable laws and regulations. The ethical, scientific, and medical appropriateness of the study, as well as substantial amendments to the protocol (Supplementary Appendix), were reviewed by the Institutional Review Board (IRB). All enrolled patents were required to provide an IRB-approved written informed consent and sign a Health Insurance Portability and Accountability Act authorization form.

### Patients

Eligible patients were adults, male, or female, between 18 and 60 years of age, with a confirmed diagnosis of schizophrenia or schizoaffective disorder in accordance with the *Diagnostic and Statistical Manual of Mental Disorders, 5th edition* *(DSM-5)*. All patients were deemed clinically stable based on the positive and negative syndrome scale (PANSS) scores with study inclusion requiring no more than moderate positive symptom scores and moderate negative symptom scores. Additionally, all patients had to be on stable doses (no more than 25% change in dose) of risperidone, quetiapine, olanzapine, ziprasidone, brexpiprazole, aripiprazole, paliperidone, or lurasidone for at least 2 months for oral formulations, or at least 3 months for depot formulations, prior to screening. While female patients had to be of nonchildbearing potential, male patients and their female spouse/partners who were of childbearing potential were required to use at least two forms of highly effective birth control starting at screening and continuing throughout the study period and 90 days post study drug administration.

Patients were excluded from this study if they had any previous exposure, or known or suspected hypersensitivity to ASP4345; history of suicide attempt or suicidal behavior within 2 years prior to screening; clinically significant liver chemistry test result; history of allergic condition; clinically significant cardiovascular, gastrointestinal, endocrinologic, hematologic, hepatic, immunologic, metabolic, urologic, pulmonary, neurologic, dermatologic, psychiatric (other than schizophrenia or schizoaffective disorder), renal and/or other major disease or malignancy; diagnosis of moderate or severe tardive dyskinesia, bipolar disorder, major depressive disorder, personality disorders, neuroleptic malignancy syndrome, or anxiety disorder; febrile illness or symptomatic, viral, bacterial, or fungal infection within 1 week prior to admission to the clinical unit (day −2); clinically significant abnormality following the investigator’s review of the physical examination, electrocardiogram (ECG), and protocol-defined clinical laboratory tests at screening or day −2; a mean pulse < 40 or more than 100 bpm; mean systolic blood pressure over 160 mmHg; mean diastolic blood pressure over 90 mmHg at screening or day −2; mean corrected QT interval using Fridericia’s formula over 440 ms (for male patients) and over 460 ms (for female patients) at screening or day −2; tested positive for alcohol or drugs of abuse (amphetamines, barbiturates, cannabinoids, cocaine, and opiates) at screening or at day −2; and/or an acute exacerbation of schizophrenia requiring hospitalization within the last 3 months or an increase in antipsychotic medication (with reference to drug or dose) within the last 4 weeks prior to screening.

### Study outcomes

Safety assessments included monitoring of adverse event (AEs) (conducted during screening period, baseline visit [defined as the mean of the scores from study day −2 pre-dose and day −1 pre-dose] and days 1, 2–6, 7–13, 14, 15–17, and 18/early discontinuation [ED], and end of study visit [ESV]), physical health examination (conducted during screening period, day 18/ED, and ESV) and mental health examination (including tests for antipsychotic drug-induced motor disorders), clinical laboratory evaluations (conducted during screening), and ECG monitoring (conducted during screening period and days 1, 7–13, 14, 15–17, and 18/ED). Mental health evaluations included abuse liability based on questions from the Addiction Research Center Inventory-49 checklist (conducted during days 1, 2–6, 14, and 15–17), full spectrum of suicidality (suicidal ideation, intensity of ideation, suicidal behavior, and actual attempts) based on the Columbia-Suicide Severity Rating Scale (C-SSRS; conducted during screening period and on days 14 and 18/ED), self-evaluation of mood based on 16 dimensions using the Bond–Lader Visual Analogue Mood Scales (conducted on days 1, 2–6, 14, and 15–17), and symptom severity of schizophrenia using the PANSS and clinical global impression (CGI) scales (conducted during screening period and on day 14).

Movement disorders (conducted during screening period and on day 14) were evaluated with the five-point Abnormal Involuntary Movement Scale to assess tardive dyskinesia, the ten-item Simpson Angus Scale to assess drug-induced neurological effects, and the Barnes Akathisia Rating Scale to assess severity of drug-induced akathisia. Psychomotor function, attention, and working memory were assessed using the Detection, Identification, and One Back Tests, respectively, from the Cogstate Brief Battery (CBB) (conducted during screening period, baseline visit, and on days 1 [pre-dose], 14, and 17) given their demonstrated sensitivity to dopamine agonists in schizophrenia [14–16]. Neurophysiological biomarkers of target engagement and early therapeutic effects included gamma-band phase-locked and evoked power in response to 40-Hz auditory steady-state stimulation, mismatch negativity (MMN), and P3a amplitudes (conducted during baseline visit, and on days 1, 7–13, and 14).

Electroencephalographic (EEG) data were continuously recorded during baseline visit, and on days 1 (pre-dose), 7, and 14 with a 64-channel Brain Products ActiChamp2 system at a sampling rate of 2000 Hz. Data processing was performed offline using Matlab, EEGlab, and BrainVision Analyzer and as per established methods [17–22]. All auditory stimuli were presented to participants at 85-dB sound pressure levels using a NeuroSig stimulation unit with custom EEG acquisition software.

The auditory steady-state response (ASSR) paradigm recorded during baseline visit, and on days 1 (pre-dose), 7, and 14 utilized 1-ms clicks presented in 500-ms trains at a frequency of 40 Hz. A total number of 250 click trains were played with an inter-train interval of 0.5 s, consistent with other studies reported in the literature [17–19]. Participants were instructed to ignore auditory stimuli while staring at a fixation cross at the center of the screen. γPL was calculated on wavelet coefficients obtained from Morlet wavelet transformation of the segmented data (frequencies represented from 5 to 95 Hz, with a total number of 45 frequency layers); γPL was estimated by extracting and averaging across the 30–50 Hz frequency layer. γPL quantifies consistency of the oscillatory phase across individual trials, ranging from 0 (purely non-phase-locked activity) to 1 (fully phase-locked activity). For statistical analyses, mean values were obtained for each of the six 100-ms windows from −100 to 500 ms, relative to stimulus onset.

A passive auditory oddball paradigm was used to elicit MMN and P3a. Consistent with Seech et al. [21], 85% of the tones were standards (50 ms, 1000 Hz, *n* = 2754) and 15% were deviants (7.5%, *n* = 243 per deviant type) that differed in stimulus duration (100 ms, 1000 Hz) or both duration and frequency (i.e., “double deviant” 100 ms, 1100 Hz). All tones had 5-ms rise/fall times and were presented with a fixed 500-ms stimulus onset asynchrony. Subjects were instructed to ignore auditory stimuli while viewing a silent movie.

For MMN and P3a, deviant-minus-standard difference waves were generated for each deviant type and low-pass filtered (20-Hz zero phase shift, 24-dB/octave roll-off). MMN and P3a were computed as the mean amplitude across 135–205 and 250–300 ms ranges, respectively.

### Statistical analysis

The sample size was based on precedent set by other clinical studies of a similar nature. The number of patients planned for this study was considered sufficient to achieve the study objectives of ASP4345 safety, tolerability, and pharmacokinetics. Assignment to treatment was done using the randomization code generated by the sponsor’s Data Science Department or designee. The safety analysis set (SAS) was used to describe all demographic and baseline characteristics and safety- and tolerability-related variables, and the pharmacodynamic analysis set (PDAS) was used for analyses of the pharmacodynamic data. The SAS consisted of all patients who received at least one dose of study drug, while the PDAS included patients from the SAS for whom sufficient pharmacodynamic data were available to facilitate derivation of at least one pharmacodynamic parameter and for whom time of dosing on the day of sampling was known. All AEs were coded using Medical Dictionary for Regulatory Activities version 18.0. A treatment-emergent AE (TEAE) was defined as any AE that started or worsened following the first administration of study drug and continued until completion of the final scheduled study procedure. The actual score and change from baseline for the CBB tests by treatment group and visit, and for the electrophysiological measures by event-related potential component, electrode/composite, treatment group, and visit, were summarized using descriptive statistics (*n*; mean, SD). A linear mixed effects model with repeated measures, assuming an unstructured covariance matrix, was used for the statistical analysis.

## Results

### Patient disposition, baseline demographics, and disease characteristics

Between March 2016 and June 2017, a total of 36 randomized patients received ASP4345 (3, 15, 50, and 150 mg; *n* = 9 for each dose), 12 patients received placebo, and all 48 patients were included in the SAS and PDAS. Two patients, one each from the 3- and 150-mg ASP4345 treatment groups, withdrew from the study owing to a family emergency; however, the patient from the 150-mg group completed the treatment and follow-up periods. Patient age ranged from 27 to 60 years, a higher proportion were male (75%), and patients were primarily Black or African American (83.3%). Body mass index ranged from 18.6 (normal) to 38.9 kg/m^2^ (obese) (Table 1). All but two patients were diagnosed with schizophrenia. One patient in the placebo group and one patient in the 3-mg ASP4345 group were diagnosed with schizoaffective disorder.

**Table 1:** Subject demographics and baseline disease characteristics.

Characteristics	Placebo *n* = 12	ASP4345 (*n* = 36)			
		3 mg (*n* = 9)	15 mg (*n* = 9)	50 mg (*n* = 9)	150 mg (*n* = 9)
Median age (range), years	52 (27–60)	49 (33–60)	53 (30–60)	53 (38–58)	38 (37–50)
Sex, *n* (%)					
Male	8 (66.7)	7 (77.8)	9 (100)	7 (77.8)	7 (77.8)
Female	4 (33.3)	2 (22.2)	0 (0)	2 (22.2)	2 (22.2)
Race, *n* (%)					
White	2 (16.7)	1 (11.1)	2 (22.2)	0 (0)	1 (11.1)
Black	10 (83.3)	7 (77.8)	6 (66.7)	9 (100)	8 (88.9)
Asian	0 (0)	1 (11.1)	0 (0)	0 (0)	0 (0)
Other	0 (0)	0 (0)	1 (11.1)	0 (0)	0 (0)
BMI (range), kg/m^2^	27.35 (23.4–38.6)	28.70 (24.3–34.3)	26.20 (20.6–38.9)	30.90 (18.6–38.6)	30.00 (20.7–38.7)
COMT genotype, *n* (%)					
Met/Met	1 (8.3)	1 (11.1)	2 (22.2)	2 (22.2)	1 (11.1)
Val/Met	6 (50.0)	4 (44.4)	3 (33.3)	3 (33.3)	6 (66.7)
Val/Val	5 (41.7)	4 (44.4)	4 (44.4)	4 (44.4)	2 (22.2)

*BMI* body mass index, *COMT* catechol-O-methyltransferase, *Met* methionine, *Val* valine.

### Safety and tolerability

Among the 36 patients who received ASP4345, 29 (80.6%) patients reported a total of 73 TEAEs, and among the 12 patients who received placebo, 9 (75.0%) patients reported 34 TEAEs. The most commonly reported TEAEs were headache and somnolence (Table 2). The frequency of TEAEs in the ASP4345 and placebo groups was comparable and, importantly, no increase in frequency was noted with increasing ASP4345 doses. The TEAEs were considered by the investigator to be mild in severity, except for one occurrence each of psychotic disorder (3-mg ASP4345) and arthralgia (placebo), which were considered by the investigator to be moderate in severity. A treatment-emergent SAE of small intestinal obstruction in one patient who received 50-mg ASP4345 on day 19 (5 days after last dose) through day 23 was considered by the investigator to be severe in nature; however, this event was deemed unrelated to ASP4345 and was conservatively managed in the hospital without surgical intervention. There were no AEs that led to withdrawal of ASP4345 and no deaths were reported throughout the duration of the study. No clinically relevant changes were observed in any of the laboratory analyses or laboratory values indicative of hepatotoxicity. No AE or C-SSRS data suggest that ASP4345 has a potential effect on (causing/triggering) suicidal ideation or behavior. No drug-related movement disturbances, neurological effects or drug-induced akathisia, or drug-related changes in symptom severity (PANSS and CGI), addiction Research Center Inventory-49 checklist, C-SSRS, and Bond–Lader Visual Analogue Mood Scales were noted during this study in patients with schizophrenia.

**Table 2:** Incidence of treatment-emergent adverse events occurring in ≥2 patients in any cohort.

TEAE, *n* (%)	Placebo *n* = 12	ASP4345 (*n* = 36)			
		3 mg (*n* = 9)	15 mg (*n* = 9)	50 mg (*n* = 9)	150 mg (*n* = 9)
Headache	4 (33.3)	0 (0)	3 (33.3)	3 (33.3)	3 (33.3)
Somnolence	1 (8.3)	2 (22.2)	1 (11.1)	2 (22.2)	2 (22.2)
Constipation	0 (0)	2 (22.2)	0 (0)	3 (33.3)	0 (0)
Back pain	0 (0)	1 (11.1)	2 (22.2)	0 (0)	1 (11.1)
Dyspepsia	1 (8.3)	0 (0)	1 (11.1)	2 (22.2)	0 (0)
Upper respiratory tract infection	1 (8.3)	2 (22.2)	1 (11.1)	0 (0)	0 (0)
Insomnia	3 (25.0)	1 (11.1)	1 (11.1)	1 (11.1)	0 (0)
Anxiety	0 (0)	0 (0)	0 (0)	0 (0)	2 (22.2)
Dizziness	2 (16.7)	1 (11.1)	1 (11.1)	0 (0)	0 (0)
Abnormal dreams	0 (0)	2 (22.1)	0 (0)	0 (0)	0 (0)

Adverse events listed are individual preferred terms.

*TEAE* treatment-emergent adverse event.

### Pharmacodynamic effects on cognition

Effect sizes for the comparison of change from baseline on each Cogstate test under ASP4345 versus change from baseline under placebo at the days 14 and 17 assessments were determined (Table 3). At the day 14 assessment, moderate to large benefits in performance were observed for all doses of ASP4345 relative to placebo for the detection task. Qualitatively similar, but quantitatively smaller, beneficial effects were observed across doses of ASP4345, relative to placebo, on the Identification Task test at the day 14 assessment. However, no systematic benefits in performance were observed across doses of ASP4345 for the One Back Test on day 14. Consideration of performance on day 17, or at 72 h after the last dose of ASP4345, showed that benefits in performance on the Detection test declined by 60–80%. Consistent with this drug, benefits in performance on the Identification Task test also declined between 30% in effect size in the 3 and 50-mg ASP4345 cohorts, and by 125% in the 150-mg ASP4345 cohort 72 h after drug withdrawal. No systemic changes in performance from days 14 to 17 were evident for the One Back Test.

**Table 3:** Mean change from baseline and effect size changes in cognitive testing scores between placebo and ASP4345 on days 14 and 17.

Cognitive test	Day	ASP4345 (*n* = 36)			
		3 mg (*n* = 9)^a^	15 mg (*n* = 9)	50 mg (*n* = 9)	150 mg (*n* = 9)^b^
Detection task	Mean (SD) change from baseline on day 14	−0.045 (0.081)	−0.025 (0.085)	−0.053 (0.066)	−0.018
	Effect size on day 14	0.820	0.577	0.993	0.589
	Mean (SD) change from baseline on day 17	−0.039 (0.103)	−0.036 (0.123)	−0.045 (0.095)	−0.026 (0.085)
	Effect size on day 17^c^	0.264	0.213	0.343	0.135
Identification task	Mean (SD) change from baseline on day 14	−0.015 (0.082)	−0.025 (0.052)	−0.017 (0.059)	−0.004 (0.061)
	Effect size on day 14	0.253	0.426	0.304	0.137
	Mean (SD) change from baseline on day 17	−0.012 (0.058)	−0.031 (0.092)	−0.016 (0.049)	−0.002 (0.074)
	Effect size on day 17^c^	0.129	0.352	0.204	−0.034
One Back task	Mean (SD) change from baseline on day 14	−0.021 (0.076)	−0.014 (0.065)	−0.054 (0.065)	−0.007 (0.033)
	Effect size on day 14	0.013	−0.056	0.354	−0.135
	Mean (SD) change from baseline on day 17	−0.003 (0.052)	0.040 (0.066)	−0.078 (0.053)	−0.013 (0.050)
	Effect size on day 17^c^	−0.253	−0.239	0.780	−0.120

The effect size was the difference between ASP4345 versus placebo and was calculated as the difference between treatments in mean change from baseline divided by pooled SD of two treatment groups (Cohen’s D). The primary outcome measure for each task was the speed of correct responses in msec. A logarithmic transformation was applied to this value in order to normalize data distributions. A lower score implies better performance.

^a^*n* = 8 on days 14 and 17; ^b^*n* = 8 on day 17; ^c^72 h after last dose.

### Pharmacodynamic effects on neurophysiologic biomarkers

**Fig. 2:**
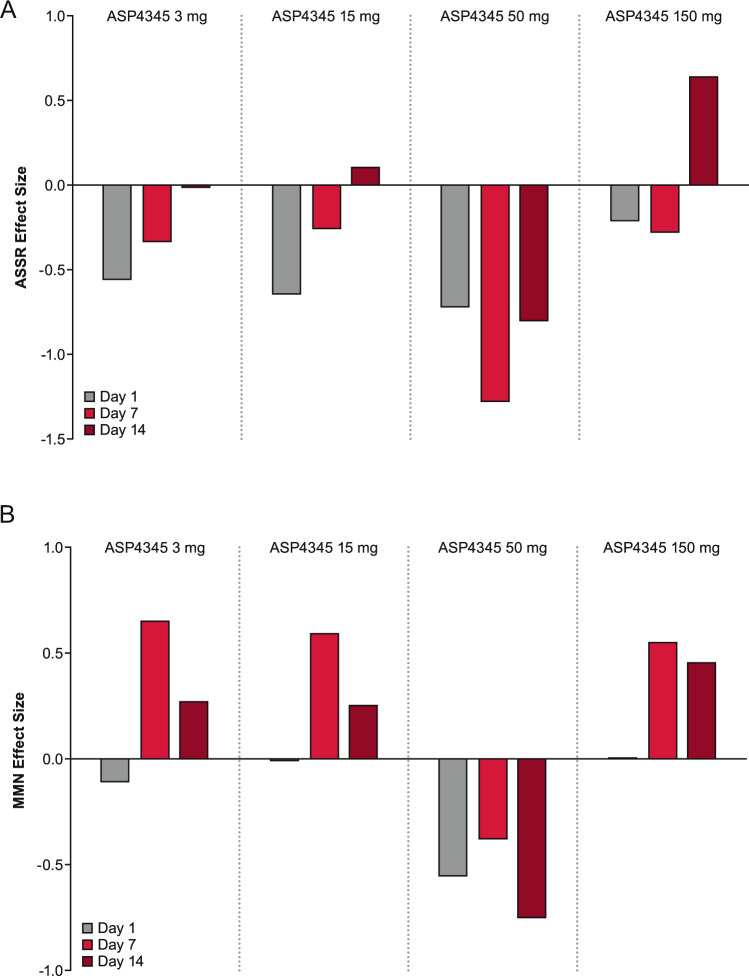
Effect size difference over time in patients treated with ASP4345 relative to placebo for **A** auditory steady-state response; **B** mismatch negativity measurements. The effects of ASP4345 (3, 15, 50, and 150 mg) on days 1, 14, and 17 (3 days after the last dose of the dopamine D_1_ receptor PAM) for the evoked electrophysiological measures of the ASSR evoked effect size (top figure) and MMN effect size (bottom figure) in patients with schizophrenia/schizoaffective disorder. The effect size of difference between ASP4345 and placebo (calculated as the difference between mean change from baseline ASP4345 and placebo, multiplied by a multiplicand (for negative peaks the multiplicand = −1 and for the positive peaks the multiplicand = 1) and divided by the pooled standard deviation for the two treatment groups) was summarized by event-related potential component, electrode/composite, and by treatment group and visit.

A positive effect was observed for ASSR on day 14 in the 150-mg ASP4345 treatment group (Fig. 2); however, this study was not sufficiently powered to detect statistically significant differences, including the 90% CI. The MMN suggests a trend for benefit with ASP4345 over placebo and these benefits were generally greatest on day 7, but levels were equivalent on day 14 for the 150-mg dose. There were no appreciable effects as compared with placebo for P3a for any treatment group.

## Discussion

The results of this phase 1 study suggest that multiple ascending oral doses of the dopamine D_1_ receptor PAM ASP4345 (3–150 mg) may be safe and well tolerated in adult patients with schizophrenia, with respect to the AE profile, lack of clinically relevant changes on laboratory tests, vital signs, ECGs, and absence of worsening of movement disorders. ASP4345 did not induce any apparent suicidality as assessed by the C-SSRS. In terms of effects on cognition, treatment with ASP4345 showed consistent and moderate benefit to psychomotor function in the schizophrenia group. A similar, but more subtle, ASP4345-related improvement was observed in visual attention. The consistent benefit across doses on these highly correlated aspects of cognition suggests that treatment with ASP4345 may provide improvements in attentional function in schizophrenia.

The hypothesis that treatment with ASP4345 improved psychomotor and attentional functions is supported by the observation that the drug-related benefits were reduced substantially once treatment was stopped (i.e., 72 h after the final dose). Performances on these same tests have been observed in patients with schizophrenia following acute and subchronic treatment with other dopamine agonists such as d-amphetamine and DAR-1001 [14, 16]. In the current study, drug-related benefits were not observed for working memory. This absence of benefit was surprising given the well-established role of dopamine neurotransmission in working memory. The measure of working memory applied here was previously shown to improve after treatment with dopamine agonists [14, 16]. Therefore, the absence of any benefit from ASP4345 treatment in this study may be due to the more complex nature of this test. A larger sample size or a longer period of treatment may be necessary to show benefits in working memory compared with the simpler measures of psychomotor function and attention. When considered together, the data from the cognitive assessments do suggest that ASP4345 enters the CNS and can positively influence dopamine-related cognitive operations.

For the EEG measures, ASSR showed an increasing effect size relative to placebo by day 14 for the 3-, 15-, and 150-mg treatment groups, while MMN showed a small to moderate effect size relative to placebo by day 7 for the 3-, 15-, and 150-mg treatment groups. The 50-mg dose did not result in improvement for either endpoint, which may be due to the low sample size of this study. The confluence of the beneficial effects on cognition and electrophysiological markers strongly suggests that treatment with ASP4345 has a facilitatory effect on dopamine neurotransmission in individuals with schizophrenia and should therefore be explored in larger studies.

An improved understanding of the dopamine D_1_ receptor role in CIAS has led to the development of other compounds to optimize the beneficial effects of dopamine D_1_ receptor stimulation in the prefrontal cortex to ameliorate cognitive deficits in patients with CIAS [5]. However, most of the currently available data for these compounds are limited to animal studies. A recently conducted phase 1b clinical trial evaluated the safety and efficacy of PF-06412562—an orally bioavailable, selective dopamine D_1_/D_5_ receptor partial agonist—for the treatment of CIAS in 95 symptomatically stable patients [23]. In this study, PF-06412562 was safe and well tolerated, but was not associated with any improvement in cognition or reward processing when compared with placebo over a 15-day treatment period. Another compound, DAR-0100A, has been clinically assessed for its effects on the schizophrenia-spectrum cognitive deficits of 16 unmedicated patients with schizotypal personality disorder using two tests of verbal working memory, the Paced Auditory Serial Addition Test and N-back test [24]. DAR-0100A was generally well tolerated, but did not provide significant benefit in attenuating working memory impairments. The authors of the DAR-0100A study emphasized the need for the development of novel dopamine D_1_ receptor agonists with improved pharmacokinetic properties, particularly with respect to half-life and oral bioavailability, and the need for a more comprehensive neuropsychological battery such as those assessed in 49 clinically stable individuals with schizophrenia treated with 0.5 or 15 mg of DAR-0100A [14]. Treatment with 15 mg of DAR-0100A showed significant effects compared with placebo on the Cogstate Schizophrenia Battery (CSSB global cognition composite score) and composite memory score, as well as on the tests for visual attention (Identification), learning (One Card Learning), working memory (One Back), and social emotional cognition.

Results of the present study support the continued use of high-density EEG recording of ASSR, MMN, and P3a as pharmacodynamic endpoints in clinical trials. These measures were selected based on several criteria described in the literature [25–28], including ease of rapid assessment with high-density montages [19, 20] for characterization of neural substrates, substantial test–retest reliability [29], relationships to cognition and daily functioning [17, 20, 30–35], and sensitivity to pharmacologic [19, 22] and non-pharmacologic interventions [36–39]/perturbations [21]. The primary limitation of this study is the small patient population because of which it is underpowered for detection of treatment-induced effects for the key physiological biomarkers or cognitive assessments. This study was not powered based on statistical consideration. The sample size was based on other clinical studies of a similar nature. However, this is a phase 1 study conducted to assess the effects of a potential drug in a small patient population and therefore no conclusion about the efficacy of ASP4345 has been drawn from the data of this study. The number of patients planned for this clinical study is considered adequate to achieve the objectives of this clinical study (adequate ASP4345 safety, tolerability, and pharmacokinetics).

In summary, ASP4345 is a novel dopamine D_1_ receptor PAM with promising pharmacokinetic (data presented elsewhere) and pharmacodynamic properties, including a trend in improvements in early auditory information processing, psychomotor function, and visual attention. The majority of TEAEs were mild, as were the drug-related AEs of constipation, headache, and somnolence. Therefore, based on the clinical meaningfulness of improving cognition in patients with schizophrenia, the current results support further evaluation of ASP4345 for the treatment of CIAS in patients with schizophrenia and schizoaffective disorder in larger randomized phase 2/3 studies.

## Funding and disclosure

This study was funded by Astellas Pharma Global Development, Inc., Northbrook, Illinois. AD, LB, RW, TU, GJM, and TZ: Astellas Pharma Global Development, Inc.—employment; LG: nothing to disclose; GAL: Heptares, NeuroSig, Astellas, NASA—personal fees, Boehringer-Ingelheim—grants. PM: Cogstate Ltd.—employment.

## Supplementary information

Supplemental Appendix

## Data Availability

Researchers may request access to anonymized participant level data, trial level data, and protocols from Astellas sponsored clinical trials at: www.clinicalstudydatarequest.com. For the Astellas criteria on data sharing see: https://clinicalstudydatarequest.com/Study-Sponsors/Study-Sponsors-Astellas.aspx.
