# Supplementary material for: Phase 1 randomized study on the safety, tolerability, and pharmacodynamic cognitive and electrophysiological effects of a dopamine D1 receptor positive allosteric modulator in patients with schizophrenia
Source: Neuropsychopharmacology. 2020 Nov 17;46(6):1145–51. doi: 10.1038/s41386-020-00908-0 (PMC8182805; doi:10.1038/s41386-020-00908-0)
Supplement: Supplementary file 1 — Supplemental Appendix [file 41386_2020_908_MOESM1_ESM.docx]

**Supplemental Appendix**

**Amendment and Administrative Change Summary**

The original protocol had two substantial amendments, which occurred prior to database hard lock. Cohort A was conducted under the original protocol, cohort B was conducted under the revised protocol incorporating Substantial Amendment 1, and cohorts C and D were conducted under the revised protocol incorporating Substantial Amendment 2.

A summary of the changes for the amendments is provided below. These changes to the protocol did not affect the overall outcome of the study.

***Substantial Amendment 1***

Substantial Amendment 1, dated 16 Jun 2016, is summarized below:

● It was clarified that abuse liability and Bond-Lader Visual Analogue Scale (VAS) would not be reviewed for dose escalation and that movement disorders would only be reviewed for dose escalation if the investigator noted a clinically relevant finding.

● It was clarified that patients would not be excluded from the study if they tested positive for benzodiazepines at screening but would be excluded from the study if they tested positive for benzodiazepines at admission to the clinical unit on Day -2.

● The interval for cognitive testing on Day 1 was updated to between 1 and 1.5 hours postdose.

● It was clarified that Day 18 assessments would be performed before patients were discharged from the clinical unit in the event of early discontinuation.

● The Positive and Negative Syndrome Scale (PANSS) profile form was added to the appendices. The Clinical Global Impression Scale (CGIS) and Columbia – Suicide Severity Rating Scale (C-SSRS) appendices were replaced with new approved versions. In addition, nonsubstantial changes included minor administrative-type changes, corrections, and clarifications.

***Substantial Amendment 2***

Substantial Amendment 2, dated 06 Sep 2016, is summarized below:

● Positive testing for hepatitis B core antibodies was removed from the exclusion criteria and serology screening. In addition, nonsubstantial changes included minor administrative-type changes, corrections, and clarifications.


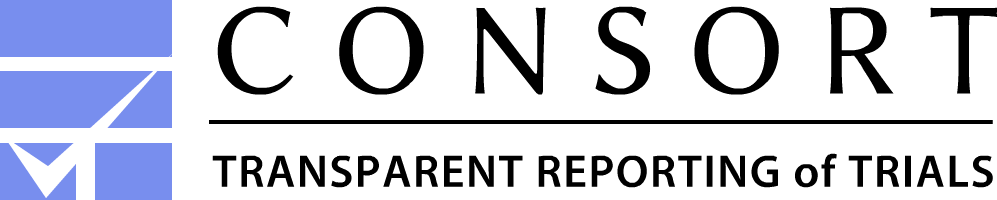


**CONSORT 2010 Flow Diagram**

## Follow-Up

Analysed (n= 12)

Analysed (n= 36)

Lost to follow-up (n= 0)

Lost to follow-up (n= 0)

## Enrollment

Allocated to Placebo (n= 12)

♦ Completed Treatment Period (n= 12)

## Allocation

Allocated to ASP4345 (n= 36)

♦ Completed Treatment Period (n= 34)

♦ Did not complete treatment period (Withdrawal by Subject, n= 2*)

*One patient re-enrolled and completed treatment and follow-up

Randomized (n= 48)

Assessed for eligibility (n=138)

## Analysis

Excluded (n= 90)

♦  Screen Failure (n= 79)

♦  Withdrawal by Subject (n= 1)

♦  Other (n= 10)
